# Unmanned Aerial Vehicle Systems for Remote Estimation of Flooded Areas Based on Complex Image Processing

**DOI:** 10.3390/s17030446

**Published:** 2017-02-23

**Authors:** Dan Popescu, Loretta Ichim, Florin Stoican

**Affiliations:** Department of Control Engineering and Industrial Informatics, University Politehnica of Bucharest, Bucharest 060042, Romania; loretta.ichim@upb.ro (L.I.); florin.stoican@upb.ro (F.S.)

**Keywords:** unmanned aerial vehicle, path planning, flood detection, feature selection, image processing, image segmentation, texture analysis

## Abstract

Floods are natural disasters which cause the most economic damage at the global level. Therefore, flood monitoring and damage estimation are very important for the population, authorities and insurance companies. The paper proposes an original solution, based on a hybrid network and complex image processing, to this problem. As first novelty, a multilevel system, with two components, terrestrial and aerial, was proposed and designed by the authors as support for image acquisition from a delimited region. The terrestrial component contains a Ground Control Station, as a coordinator at distance, which communicates via the internet with more Ground Data Terminals, as a fixed nodes network for data acquisition and communication. The aerial component contains mobile nodes—fixed wing type UAVs. In order to evaluate flood damage, two tasks must be accomplished by the network: area coverage and image processing. The second novelty of the paper consists of texture analysis in a deep neural network, taking into account new criteria for feature selection and patch classification. Color and spatial information extracted from chromatic co-occurrence matrix and mass fractal dimension were used as well. Finally, the experimental results in a real mission demonstrate the validity of the proposed methodologies and the performances of the algorithms.

## 1. Introduction

In the repertory of natural disasters, floods often cause the greatest material damage [1]. For example, floods in 2013 constituted 31% of the economic losses resulting from natural disasters [2]. Therefore, the forecasting, prevention, detection, monitoring, and flood damage assessment are of paramount importance for public authorities and people. Because early warning is essential for limiting the loss of life and property damage, many works examine real time flood detection systems [1,3,4]. For example, the integration of several existing technologies was used in Taiwan to develop a coastal flooding warning system [3].

The problem that we are solving in this paper is the evaluation of small flooded areas in rural zones with the aid of a cheap solution based on processing of images taken by the unmanned aerial system designed by the authors. The result is necessary to evaluate the post-flood damage by the local authorities (to determine relief funds) and assurance companies (to determine payments). Even small flooded areas are investigated and classified. For this purpose two sub-problems must be solved. First is the optimal coverage of the area to be monitored, from the point of view of energy consumption (a limiting factor, especially if the UAV is electrically powered through a battery) and trajectory length. The second sub-problem is the detection and estimation in terms of flooded areas of the covered regions.

For the purpose of flood detection and monitoring, different sensors have been used: optical sensors, multi-spectral sensors and synthetic aperture radars (SARs). Satellite remote sensing imagery offers less spatial and temporal resolution than aircraft and UAVs, but a larger field of view. It was successfully used on large-scale geographic analysis on the flood overflow area. For example, images from Landsat Thematic Mapper/Enhanced Thematic Mapper Plus (TM/ETM+) sensors were used to monitor the floods near Lena River (Siberia) with a spatial resolution of 30 m and a temporal resolution of 2.6 days [5]. On the other hand satellite images in the visible and near infrared spectrum are highly dependent on cloud conditions whereas radar transmitters and receivers can be used independently of weather conditions [6]. Based on the surface water extent, measured with a microwave remote sensor (Radiometer for Earth Observation System AMSR-E and AMSR2), the authors in [7] implemented a method for detecting floods at large scale. In [8] the authors propose to combine aerial thermal data with high resolution RGB images in order to quickly and accurately identify the sub fluvial springs of a stream. Both cameras, thermal and action, are installed on a rotor platform which is able to support more weight, but has a smaller surveillance area.

Combining information from space, aerial and ground [6], an integrated system using different technologies (remote sensing, the Global Navigation Satellite System (GNSS), data transmission, and image processing) was implemented in China for monitoring and evaluating flood disasters. Because of the high cost, aircrafts use SAR only for serious and urgent flood cases. The spatial resolution is of 3 m at 9 km swath and the monitoring is in real time, independent of weather. Among the methods for flood detection, the interpretation of optical remote images is widely used and also gives the best results concerning price and accuracy.

In order to detect the flood by image analysis, three solutions usually appear in the literature: (a) use of images from satellites [9,10,11]; (b) use of images from fixed cameras on the ground [4,12,13]; and (c) use of images from aircrafts or UAVs [14].

The Advanced Land Observing Satellite Phased Array type L-band Synthetic Aperture Radar (ALOS PALSAR) satellite [11] provides multi-temporal data which maps large zones of flood via a classification into flood and non-flood areas. Based on this classification and on images taken at pre-flood and post-flood time instants, information about the flooding hazard is provided. In general the satellite applications for flood detection, like those presented in [10] from Spot-5 imagery, or in [15] by WorldView-2 satellite imagery, are based on high spatial resolution images and have the disadvantage of being high-cost solutions, hence less approachable for public use. In addition, these solutions have the disadvantage of being sensitive to weather patterns (clouds or other obscuring weather features will render them useless).

An alternative approach for monitoring flood disasters is the system of fixed cameras proposed in [12] which is based on the dynamic detection of floods via intrusions of objects in the video frames. These objects are separated by segmentation from the rest in the image.

To monitor and evaluate the flood disasters, concatenated images, created by photomosaic generation, can be useful. Thus, the gaps or duplications of flooded regions, in different analyzed images, are avoided. In this case, the UAV solution is a cheaper and more flexible one which can ensure superior image resolution even under adverse weather conditions. In this direction, the authors in [16] developed a solution for detection and evaluation of the dynamic evolution of the flood based on a collaborative team of UAVs. More recently a multicopter-based photogrammetry procedure was used to evaluate the effect of an earthquake on complex architectural landscapes [17]. Also, Feng et al. [18] used a UAV for urban flood monitoring and showed that such platforms can provide accurate flood maps. In their proposed method, the authors show how the acquired images are ortho-rectified and combined into a single image. Subsequently, the flood detection is realized through feature extraction from gray co-occurrence matrix and forest tree classifier methods. Boccardo et al. [19] compare the main advantages/disadvantages of fixed-wing UAV versus rotor platforms for area surveillance. So, rotor platforms can be used only for very small areas or isolated buildings, while for small and medium areas fixed-wing UAVs are recommended. For large areas, UAV teams with pre-positioned stand-by can successfully perform the aerial surveillance of the disaster affected areas. Systems using UAVs are able to operate at lower altitude and to acquire multi-view, repetitive images with high resolutions [20]. These systems (fixed-wing type) are used to provide large image blocks to perform an image-based registration on multi-temporal datasets.

Control of a team of collaborative agents (UAVs in our case) is challenging, especially so under external perturbations, loss or delay of communication, etc. Therefore, the usual approach is to have a hierarchical control structure: the lower-level controller (the “autopilot” implemented on-board tracks a given reference) and the higher-level controller (“mission flight management”) provides a reference trajectory [21].

Any mission has specific design requirements for the trajectory generation procedure [22,23]. Foremost in observation missions (surveillance, photogrammetry, target tracking, etc.) is to maintain a constant velocity or to allow variation within a narrow band (such that the photos taken cover uniformly the area under observation [24]). Whenever a team of UAVs is considered, additional issues appear (e.g., collision and avoidance constraints [25]). Not in the least, the trajectory has to be feasible (in the sense that it respects the UAV dynamics) Additional limitations on trajectory length, obstacle and collision avoidance are also encountered. 

A promising framework is the flat representation of dynamical systems [26]. This approach expresses the states and inputs independently though a so-called flat output (which “hides” the underlying link between the states and inputs). Relatively recent work, has concentrated on providing flat characterizations which handle well numerical issues and have a manageable complexity [27,28]. In this sense, B-spline functions are an interesting choice: their geometrical properties lead to a good flat output parametrization and allow construct optimization problems which integrate costs and constraints in order to obtain the desired results [29]. Assuming that all low-level control loops are already designed such that a predefined trajectory is followed accurately and the payload is stabilized, we can reduce the path generation problem to an optimization problem where various constraints, parameters and costs are taken into account. To conclude, a flat representation which accounts for the low-level dynamics of the autopilot (approximated by first and second-order dynamics) and uses the properties of B-spline basis functions will provide a comprehensive and flexible framework [30]. In particular, it is possible to penalize trajectory length and energy costs, guarantee obstacle avoidance and pass through or within a pre-specified distance from a priori given way-points. 

In order to detect and segment the flooded regions from the acquired images, texture and color analysis can be employed. Texture analysis techniques are a subject extensively studied in literature [31,32,33,34], but all suggested solutions are tailored to the specific context of the application considered. Moreover, there is a substantial interest in studying methods using the grey level co-occurrence matrix for texture characterization, but extremely little work is done when multi-spectral (color) co-occurrence is concerned [33,34]. All the above image acquisition strategies impose strict constraints on the photographs’ capture during the UAV mission, i.e., photographs have to be captured: at a constant height (low/medium/high—the classification is relative, depending on context and application); such that there is a predefined overlap between neighboring photographs and there are no gaps in the area of interest (such that a photo-mosaiced image covering the entire area is computed). While there are many specialized software applications which can merge photographs with partial overlap to generate a continuous mapping and detect features of interest, there are still open issues in the generation of the flight path to be followed by a UAV [22]. This apparently simple problem has a number of intricacies: turn maneuvers of the UAV should not “cut” into the shape of the area under observation, maximal distance between consecutive path lines has to be respected and, not in the least, the UAV operational costs (energy, time of travel) should be minimized [23,24].

In this paper, we implemented new solutions concerning the system concept, path generation and image segmentation. As first novelty, we propose a multilevel system, with two components, terrestrial and aerial, as support for image acquisition and transmission from a specified region. The terrestrial component comprises a Ground Control Station (GCS, as coordinator or master node at distance), more Ground Data Terminals (GDTs, as a fixed nodes network for data acquisition and transmission), and a launcher. The aerial component contains mobile nodes (UAV—fixed wing type). The communication is established via internet (GDTs—GCS) or direct radio (in rest). This hybrid network has the advantage of extending operational area. The fixed-wing type UAV for image acquisition was developed by the authors in the Multisensory Robotic System for Aerial Monitoring of Critical Infrastructures (MUROS) project [35] funded by the Romanian National Research Program Space Technology and Advanced Research (STAR) from the Romanian Space Agency (ROSA) [36]. The proposed system is completely autonomous, apart from the take-off stage where a human operator is needed, and can be monitored and controlled at distance from the operational field. The area to be monitored is covered with the aid of a trajectory designed by a suitable optimization problem while the acquired images are analyzed in order to detect and assess the extent of floods. The second novelty refers to previous trajectory design implementations. So, the main contributions are: (a) the full dynamics (GCS + autopilot levels)—described in the flat representation and (b) the area under surveillance—partitioned between UAVs, such that the workload is balanced and collision with another UAV is impossible. The third novelty is a new solution for detection and quantitative evaluation of flooded small areas, based on the gliding box algorithm and local image processing. The advantages of this solution are the following: lower cost compared to a manned aircraft or a satellite solution, better resolution than a satellite solution, and the possibility of operating on cloudy conditions. The proposed method simultaneously uses pixel distribution and color information taking into account the chromatic co-occurrence matrix and mass fractal dimension on color components. The features used are not fixed as in [18] but rather they are being adapted to each application and environment condition. Results of the feature selection (especially associated with color channels) eliminate the temporal (colors of vegetation) and geographical influences (soil and vegetation colors, buildings and infrastructures).

The rest of the paper is organized as follows: in Section 2, first, the model of the UAV system based on hybrid wireless network is presented and second, the methodology and algorithms for image processing with the aim of flooded area detection, segmentation and estimation are described and implemented. The results obtained from images acquired with a fixed-wing UAV, designed by a team including the authors, are reported and analyzed in Section 3. For image acquisition, a path generated by the method introduced in Section 2 is used. Finally, the conclusions and discussions are reported in Section 4 and, respectively, Section 5.

## 2. Instruments and Methods

It is difficult and expensive to obtain precise data of the flood size within a certain small area from aerial photographs. As it was stated in Section 1, in this paper we propose a cheap and accurate solution to estimate the size of the dispersed small flooded areas. The solution is based on image segmentation obtained by a hybrid aerial—ground network integrated in internet. Three important sides are investigated: (a) the configuration of the network (which was partially implemented in the MUROS project [35] and will be finalized in SIMUL project [36]); (b) the trajectory control; and (c) the image processing for flooded area detection and estimation. The entire system is monitored and controlled remotely by GCS, via the Internet. 

### 2.1. UAV System

**Figure 1:**
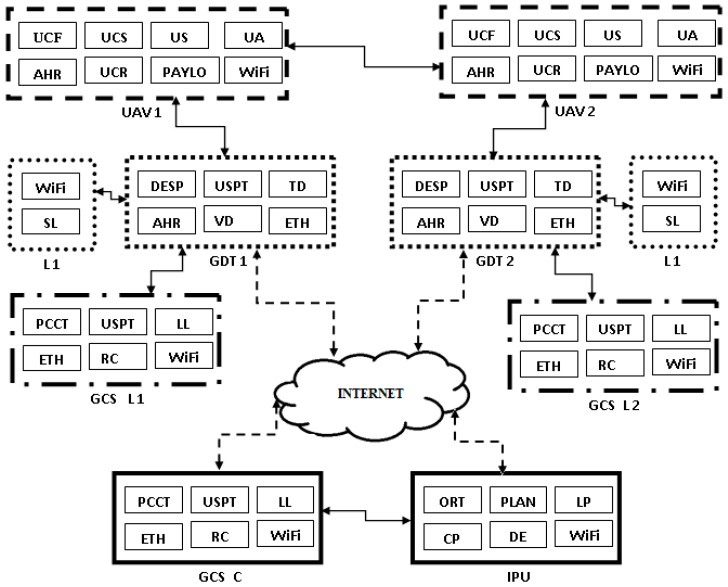
Block diagram of the system.

**Figure 2:**
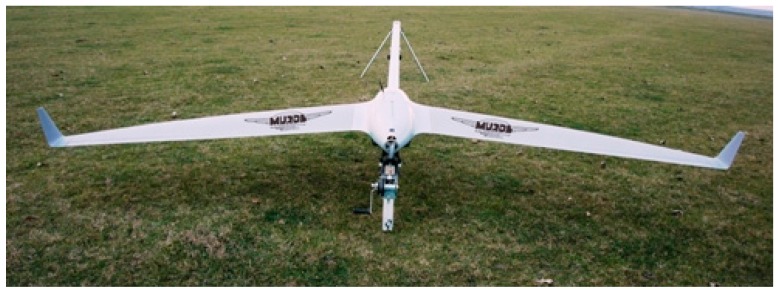
UAV MUROS on launcher.

**Figure 3:**
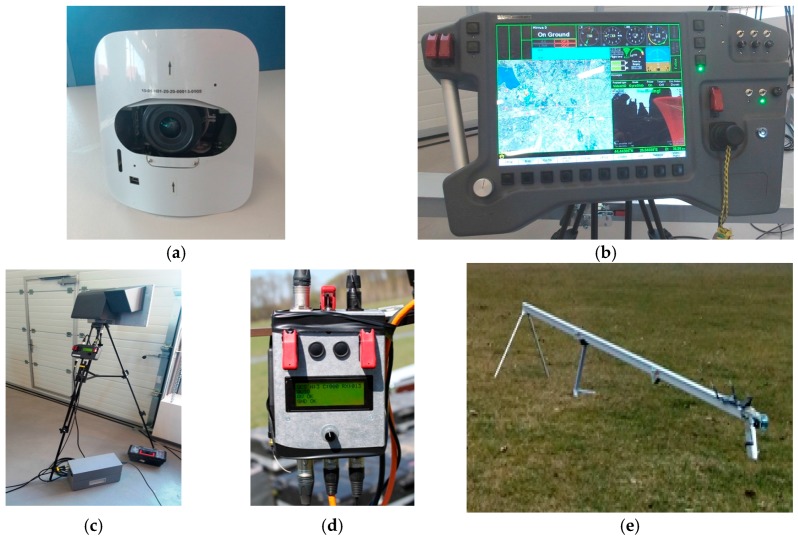
System components: (**a**) Payload photo; (**b**) GCS; (**c**) GDT; (**d**) ID box; (**e**) Launcher.

The proposed system is configured as a hybrid network both with fixed nodes (terrestrial part) and mobile nodes (aerial part). The terrestrial part consists of the following components, which are considered at fixed locations during the mission (Figure 1): Ground Data Terminals (GDTs), Launchers (Ls), Ground Control Station (GCS) and Image Processing Unit (IPU). The aerial part contains mobile nodes (UAVs, fixed wing type) which fly over a specified flooded zone. GCS is located at distance from the operational field and the communication is made via GSM + Internet. Four wireless communication channels were used: GCS-GDT (GSM + internet), UAV-GDT (radio) and L-GDT (radio), and UAV-UAV (radio). GDT-GCS connection uses a modem GPRS/LTE as router via Ethernet interface. It is a Virtual Private Network. The block diagram of the system consists of several modules, wired to a common control bus. Each module contains a Central Processor Unit (CPU), a Power Supply Unit (PSU), and a Controller Area Network (CAN) adaptor. The wireless module is characterized by the following: (a) radio modem; (b) frequency: telemetry—[3.3 GHz–3.5 GHz], video—2.4 GHz; (c) Data rate: telemetry—230 kbps, video—analog PAL; (d) range: telemetry—20 km, video—15 km. The significance of the module abbreviations in Figure 1 and their functions are presented in Table 1. Figure 2 and Figure 3 present the principal components of UAV system, used for flood detection: UAV MUROS, GCS, GDT with ID box, Payload with camera, and Launcher. 

### 2.2. Trajectory Control

**Figure 4:**
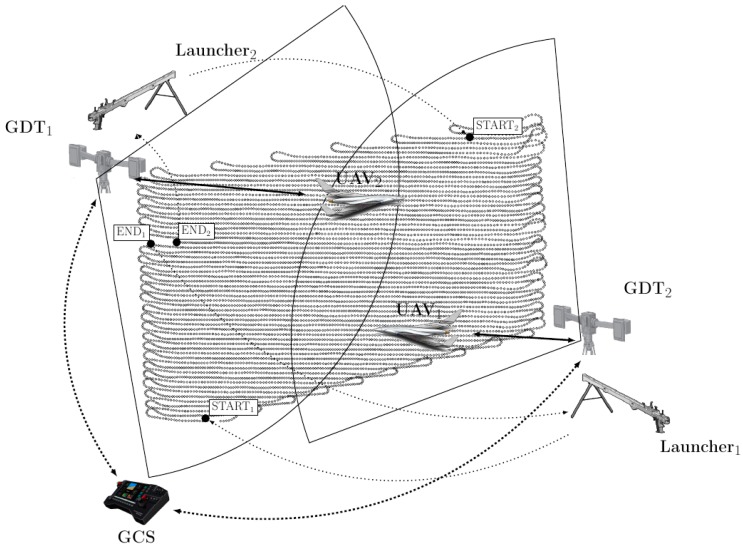
Model for trajectory generation in two-UAV applications.

For image acquisition, the UAVs must follow specific trajectories such as simultaneously cover the monitored area (Figure 4). As stated earlier, we propose to use flat output characterizations to describe the dynamics of the UAVs and further use B-spline parameterizations of the flat output in order to enforce various constraints and penalize some desired cost in the resulting constrained optimization problem. 

Let us consider the nonlinear dynamics in standard notations [37]:

(1) $$x^{\prime }(t)=f(x(t),u(t))$$

where $x(t)\in R^{n}$ is the state vector and $u(t)\in R^{m}$ is the input vector. The system (1) is called differentially flat if there exists $z(t)\in R^{m}$ such that the states and inputs can be expressed in terms of z(t) and its higher-order derivatives:

(2) $$\begin{array}{l} x(t)=\Theta (z(t),z^{\prime }(t),...,z^{\left(q\right)}(t))\\ u(t)=\Phi (z(t),z^{\prime }(t),...,z^{\left(q+1\right)}(t)) \end{array}$$

where $z(t)=Y(x(t),u^{\prime }(t),...,\;u^{\left(q\right)}(t))$.

Further, let us consider the simplified UAV dynamics with north, east, down directions ($p_{n}$, $p_{e}$ and *h*) and yaw angle ψ as states:

(3) $$\begin{array}{ll} {p^{\prime }}_{n}=V_{a}\cos\psi \cos\gamma , & {p^{\prime }}_{e}=V_{a}\sin\psi \cos\gamma \\ h^{\prime }=V_{a}\sin\gamma , & \psi^{\prime }=\frac{g}{V_{a}}\tan\varphi \end{array}$$

The autopilot is assumed to control directly the fligth-path angle γ, airspeed $V_{a}$ and roll angle φ through input elements $\gamma^{c}$, ${V_{a}}^{c}$ and $\varphi^{c}$, respectively:

(4) $$\gamma^{\prime }=b_{\gamma }(\gamma^{c}-\gamma ),\;{V_{a}}^{\prime }=b_{V_{a}}({V_{a}}^{c}-V_{a}),\;\varphi^{\prime }=b_{\varphi }(\varphi^{c}-\varphi )$$

with parameters $b_{\gamma }$, $b_{V_{a}}$ and $b_{\varphi }$ accordingly chosen. Note that the closed-loop dynamics of the autopilot are simplified to first-order dynamics (a reasonable assumption in many circumstances).

Using the flat output $z={\left[z_{1}\;z_{2}\;z_{3}\right]}^{T}={\left[p_{n}\;p_{e}\;h\right]}^{T}$ we may express the dynamics in their flat representation as follows:

(5) $$\begin{array}{ll} \psi =\arctan\frac{{z^{\prime }}_{2}}{{z^{\prime }}_{1}}, & V_{a}=\sqrt{{z^{\prime }}_{1}^{2}+{z^{\prime }}_{2}^{2}+{z^{\prime }}_{3}^{2}}\\ \gamma =\arctan\frac{{z^{\prime }}_{3}}{\sqrt{{z^{\prime }}_{1}^{2}+{z^{\prime }}_{2}^{2}}}, & \varphi =\arctan\left(\frac{1}{g}\cdot \frac{{z^{''}}_{2}{z^{\prime }}_{1}-{z^{''}}_{1}{z^{\prime }}_{2}}{\sqrt{{z^{\prime }}_{1}^{2}+{z^{\prime }}_{2}^{2}+{z^{\prime }}_{3}^{2}}}\right) \end{array}$$

together with the auxiliary elements

(6) $$\begin{array}{l} V_{a}^{c}=\sqrt{{z^{\prime }}_{1}^{2}+{z^{\prime }}_{2}^{2}+{z^{\prime }}_{3}^{2}}+\frac{1}{b_{Va}}\cdot \frac{{z^{\prime }}_{1}{z^{''}}_{1}+{z^{\prime }}_{2}{z^{''}}_{2}+{z^{\prime }}_{3}{z^{''}}_{3}}{\sqrt{{z^{\prime }}_{1}^{2}+{z^{\prime }}_{2}^{2}+{z^{\prime }}_{3}^{2}}},\\ \gamma^{c}=\arctan\frac{{z^{\prime }}_{3}}{\sqrt{{z^{\prime }}_{1}^{2}+{z^{\prime }}_{2}^{2}}}+\frac{1}{b_{\gamma }}\cdot \frac{{z^{''}}_{3}\left({z^{\prime }}_{1}^{2}+{z^{\prime }}_{2}^{2}\right)-{z^{\prime }}_{3}\left({z^{\prime }}_{1}{z^{''}}_{1}+{z^{\prime }}_{2}{z^{''}}_{2}\right)}{\left({z^{\prime }}_{1}^{2}+{z^{\prime }}_{2}^{2}+{z^{\prime }}_{3}^{2}\right)\sqrt{{z^{\prime }}_{1}^{2}+{z^{\prime }}_{2}^{2}}},\\ \varphi^{c}=\arctan\left(\frac{1}{g}\cdot \frac{{z^{''}}_{2}{z^{\prime }}_{1}-{z^{''}}_{1}{z^{\prime }}_{2}}{\sqrt{{z^{\prime }}_{1}^{2}+{z^{\prime }}_{2}^{2}+{z^{\prime }}_{3}^{2}}}\right)+\frac{1}{b_{\varphi }}\cdot \frac{1}{1+{\left(\frac{1}{g}\cdot \frac{{z^{''}}_{2}{z^{\prime }}_{1}-{z^{''}}_{1}{z^{\prime }}_{2}}{\sqrt{{z^{\prime }}_{1}^{2}+{z^{\prime }}_{2}^{2}+{z^{\prime }}_{3}^{2}}}\right)}^{2}}\cdot {\left(\frac{1}{g}\cdot \frac{{z^{''}}_{2}{z^{\prime }}_{1}-{z^{''}}_{1}{z^{\prime }}_{2}}{\sqrt{{z^{\prime }}_{1}^{2}+{z^{\prime }}_{2}^{2}+{z^{\prime }}_{3}^{2}}}\right)}^{\prime } \end{array}$$

The major difficulty lies in the fact the constraints and costs are expressed as functions of state and input which do not necessarely translate well in formulations involving the flat output *z*. The usual solution is to parametrize the flat output after some basis functions ($B_{d,i}(t)$):

(7) $$z=\underset{i}{\Sigma }\;P_{i}B_{d,i}(t)=PB_{d}(t),$$

and to find the parameters $P_{i}$ which are, in some sense, feasible and optimal. Here, the parameter d denotes the degree of the B-spline functions. That is, each B-spline function can be seen as a piecewise combination of polynomial terms of degree d. Due to the particularities of the construction, a B-spline function of order d is continuous at least up to its (d-1) derivative. B-splines, due to their properties [30], permit to express the constrained optimization problem in terms of their control points $P_{i}$ (grouped here in column form in matrix *P*):

(8) $$\begin{array}{l} P^{*}=\arg\min\;\int_{t_{0}}^{t_{N}}\left||\Xi (B_{d}(t),P)|\right|dt\\ \mathrm{subject}\;\mathrm{to}\;\Psi_{1}(B_{d}(t),P)=0,\;\Psi_{2}(B_{d}(t),P)<0 \end{array}$$

where mappings $\Xi (B_{d}(t),P),\;\Psi_{1}(B_{d}(t),P),\;\Psi_{2}(B_{d}(t),P)$ are short-hand notations which denote the cost, equality and inequality constraints, respectively. The cost can impose any penalization we deem necessary (length of the trajectory, input variation or magnitude, etc.) and constraints cover way-point validation, input magnitude constraints, etc. In general, a problem like Equation (8) is nonlinear and hence difficult to solve (particular solutions exploit the geometrical properties of the B-spline functions and/or heuristic methods).

Considering multiple UAVs further increases the difficulty of the problem. In particular, we need to decide how the way-points are partitioned between the UAVs. One, rather cumbersome, solution is to attach to each way-point a binary variable and force that at least one of the UAVs passes through it. In practice, this can be relaxed, without any loss of generality to a condition which assumes that each UAV covers a contiguous part of the surveilled region. Moreover, it makes sense to partition the regions into areas of equal length parallel with the direction of travel. Then, each UAV has to cover its own independent region with additional collision avoidance constraints which may become active around the edges (since the UAV make turns which get out from under their surveillance area). To cover this possibility we may consider collision avoidance at the autopilot level (proximity sensors) or, more robustly, at the GCS level by either introducing additional constraints in the trajectory design procedure or, preferably, by changing the start and end points for each of the agent (such that neighboring points are reached at different moments in time).

### 2.3. Image-Based Flood Detection System

In order to fulfill the mission of detection, segmentation and estimation of the flooded areas, successive images are taken with constant rate on the pre-determined trajectory, like in the above section. For flooded area estimation, a patch-based segmentation was used. So, each image is partitioned in small boxes (e.g., in our application, patches of dimension 50 × 50 pixels), using a partitioning algorithm of images [38]. Note that the patch dimension is chosen depending on the image resolution and the texture of the segmented RoI (in our case, the flood). From a cluster of such patches (boxes), manually selected, a group of efficient features for flood detection is established based on a performance indicator. The features are used to create two classes: flood class (*F*) and non-flood class (*NF*). The propose method for image processing and interpretation has two phases: the learning phase and the mission phase. Both the learning images and test images were captured by the same camera device. Because the characteristics of the flood images can differ for each application, the learning phase is necessary to establish the class representatives and the signature patch structure. In the mission phase, a trajectory covering the investigated area is established. The acquired images are concatenated and processed to create an orthophotoplan without overlapping and without creating gaps. To this end, an overlap of 60% between two adjacent images is necessary to create an orthophotoplan. Then, they are indexed with an ID number in chronological order and are partitioned in the same way as in the learning phase. Based on the features selected in the learning phase, a similarity criterion is used to assign each patch to the class *F* or *NF*. Finally (estimation step), on one hand each patch of *F* is marked with white and is returned to the initial image, and, on the other hand a binary matrix of patches (BMP) is created with logical 1, if the correspondent patch belongs to *F*, and 0, in rest. By counting the “1”s from BMP, taking into account the total number of patches, the relative flooded area is evaluated.

The image characteristics may change as a function of distance from the ground and camera inclination with respect to the vertical axis. To avoid such issues the UAV has to respect a few additional constraints: (a) the altitude remains constant (even through some ground areas, may have different heights, we take as reference the water level, which remains constant). Floods are approximately at the same distance from the UAV, hence, if the flight plan is accurately followed, the resolution remains approximately the same for a given reference altitude; (b) the payload camera has to be oriented such that the lens are perpendicular to the surface of the Earth.

For each UAV there is a channel in GCS for image acquisition and, at the end of the mission, the images from all the UAVs are stored and processed in IPU. The methodology for flood evaluation based on patch analysis consists in the following steps:

1. In IPU, ortho-rectified images are created and then they are combined into a single image without overlapping and without gaps (orthophotoplan).
2. From the orthophotoplan, adjacent cropped images of dimension 6000 × 4000 pixels are investigated for flood evaluation.
3. non-overlapping box decomposition of the tested image is made. So, a grid of boxes is created and its dimension will represent the resolution of flood segmentation. Thus, if the image dimension is $R\times M=2^{r}\times 2^{m}$ and the box dimension is $2^{u}\times 2^{v}$, then the resolution of segmentation (BMP dimension) is $2^{r-u}\times 2^{m-v}$.
4. The flood segmentation is made by patch classification in two regions of interest (flood—*F* and non flood—*NF*) taking into account the patch signatures and class representatives, which contain information about color and texture. As we mentioned earlier, the process has two phases: the learning phase (for feature selection and parameter adjustment) and the mission phase (for flood detection, segmentation and evaluation). Flood evaluation is made for each cropped image and, finally, the sum of partial results is calculated.

#### 2.3.1. Learning Phase

Generally, the aerial images taken from UAVs have textural aspects. Moreover, the remote images for water (and particularly for flood) are characterized by high contrast in texture behavior between the flooded zones and the remaining soil. Therefore, the texture information and, in particular, texture features can be used for flood detection. The selection of effective features must group the patches with flood and differentiating them from the non-flood ones (it must increase the between-class separability and decrease the within-class variance). To this end, a set of significant texture features were analyzed in the learning phase, in order to select the most efficient ones for the classification process. The tested features are of different types: mean intensity (*Im*), contrast (*Con*), energy (*En*), entropy (*Ent*), homogeneity (*Hom*), correlation (*Cor*), variance (*Var*), mass fractal dimension (*Dm*), lacunarity (*L*) and histogram of Local Binary Pattern (*LBP*). They take into account the chromatic information as well (on R, G, B, H, S and V color components). The general formulas for the most used features in texture classification are given in Table 2, where: *R* is the number of rows of the image representation (matrix *I*), *M* is the columns of *I* and *K* represents the levels on color channels. *C_d_* is the normalized co-occurrence matrix [38] calculated as an average of the co-occurrence matrices *C_d_*,*_k_* taken on eight directions, *k* = 1, 2, …, 8 (for *θ* = 0°, 45°, 90°, 135°, 180°, 225°, 270° and 315°, respectively) at distance *d* (in pixels). The notations: *H*_0_, *H*_1_, …, *H*_s−1_ represents the components of *LBP* histogram [39]. *Dm* (15) is calculated, based on Differential Box-Counting (DBC), for monochrome images, in [40]. A grid of boxes is created with the image divided in boxes with the factor *r*. For a box in position (*u*, *v*), the difference *n_r_*(*u*, *v*) between the maximum value *p*(*u*, *v*) and minimum value *q*(*u*, *v*) of the intensity are considered. Then, the sum of all the differences (17) is used to evaluate *Dm*. Similarly, the lacunarity *L*(*r*) is calculated as in [38].

To evaluate the characteristics derived from co-occurrence matrix, besides the classical gray level co-occurrence matrix [33], applied on each color channel, we used the mean color co-occurrence matrix (CCM), between pairs of two spectral components of an input image [41]. So, in *H*, *S*, *V* decomposition, the image *I* is seen as a three-dimensional array with *R* rows, *M* columns and 3 layers (spectral bands). Each array element can take *L* positive integer (discrete values representing the color component’s intensity of each pixel). The image *I* can be mathematically defined as: $I\in N^{R\times M\times 3}$).

Let *H* and *S* two components of a color space *H*, *S*, *V*. So, the mean CCM is considered as a square matrix, having L×L elements in *N*. It has two parameters: the distance *d* (the co-occurrence is the same as in GLCM case), and the component-pair (*H*, *S*) between which it is calculated. Each element of the mean color co-occurrence matrix $CMM_{d}^{HS}(i,j)$ represents how many times a pixel of the *H* component, having an intensity level of *i*, is located near a pixel with intensity *j* in the spectral component *S*, at a *d* distance. Then, the elements of the mean CCM are [37]:

(9) $$CCM_{d}^{HS}(i,j)=\frac{1}{8}\sum_{x=0}^{n-1}\sum_{y=0}^{m-1}\left\{\begin{array}{ll} 1,\mathrm{if}H(x,y)=i & \mathrm{and}S(x+d,y+d)=j\\ 0, & \mathrm{otherwise} \end{array}\right\}$$

Obviously, the next symmetry can be easily demonstrated:

(10) $$CCM_{d}^{HS}={\left[CCM_{d,k+4}^{SH}\right]}^{T},\;k=1,2,3,4$$

**Figure 5:**
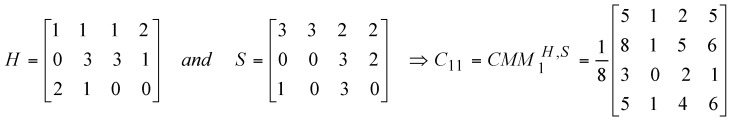
Example of calculating mean CMM.

A simple example of calculating the mean CCM is given in Figure 5, where we consider two image components $H,S\in N^{3\times 4}$, having 4 levels of pixel intensity, and the mean *CCM* computed between these two components, along a distance *d* = 1:

The algorithm for calculating CMM is presented [41] and the pseudocode in Appendix A. In order to establish the features to be selected, a cluster of 20 patches containing only flood (*PF*) are considered to form the representatives of the class “flood” (*F*) and 20 patches containing non flood elements (*PNF*), e.g., buildings and vegetation, are considered for the class “non flood” (*NF*). Each candidate feature *T_i_* to flood signature is investigated according to the following algorithm:

- *T_i_* is calculated for all the learning patches (*PF*) and the confidence interval $[m_{i}-3\sigma_{i},m_{i}+3\sigma_{i}]={ℑ}_{i}$ is determined, where $m_{i}$ and $\sigma_{i}$ represents, respectively, the mean and the standard deviation of *T_i_*.
- Similarly, *T_i_* is calculated for all the learning patches from *PNF* and the resulting set of values is noted as *NF_i_*.
- A confidence indicator for feature *T_i_*, $CI_{i}$ is created:
  (11) $$CI_{i}=\left\{ \begin{array}{l} 1,\;if\;{ℑ}_{i}\cap NF_{i}=\varphi \\ 1-\frac{\eta (\lambda_{i})}{\eta (PNF)},\;if\;{ℑ}_{i}\cap NF_{i}=\lambda_{i} \end{array}\right.$$
  where, η(*A*) is the cardinal number of the set *A*.
- The features *T_i_* with greatest $CI_{i}$ are selected in decreasing order, until the fixed number of features imposed for flood signature is reached. For example, in Section 3, a signature *T*, with 6 elements is considered (12):
  (12) $$T=\left[T_{1},T_{2},T_{3},T_{4},T_{5},T_{6}\right]$$
- As a consequence of the signature *T*, a set ℑ of confidence intervals is created (13). ℑ will be the representative of the class *F*:
  (13) $$ℑ=\left[{ℑ}_{1},{ℑ}_{2},{ℑ}_{3},{ℑ}_{4},{ℑ}_{5},{ℑ}_{6}\right]$$
  where:
  (14) $${ℑ}_{i}=\left[m_{i}-3\sigma_{i},m_{i}-3\sigma_{i}\right]$$
- For each selected feature *T_i_* a weight *w_i_* is calculated as follows. Another set of 100 patches (50—flood and 50—non flood) is considered and the confusion matrix for the feature *T_i_* is calculated based on a preliminary classification criterion: the patch B∈F if $T_{i}\in {ℑ}_{i}$.

The weight *w_i_* is established as in Equation (15):

(15) $$w_{i}=\frac{F,F+NF,NF}{F,F+F,NF+NF,NF+NF,F}$$

where *F*,*F* represents the number of patches manually selected as belonging to class *F* and classified to class *F* after feature *T_i_*. Similarly, *F*,*NF* represents the number of patches manually selected as belonging to class l*F* and classified to class *NF* after feature *T_i_*.

Observations:

- Obviously, $CI_{i}=1$ represents an ideal situation and are not encountered.
- If $\lambda_{i}=\lambda_{j}$, then *T_i_* and *T_j_* are redundant and one can be eliminated.

#### 2.3.2. Mission Phase

In the mission phase, the images from orthophotoplan are decomposed in patches with dimension of 50 × 50 pixels. Each patch (box) is indicated by a pair (row number, column number) in the squared grid of the image with an ID number. The mission phase has three steps: patch classification, image segmentation and flood estimation. 

For classification of a box (*B*) of as flooded, a weighted vote *D* is considered (16), where *D*(*B*) is the sum of partial weighted vote for each selected feature (17):

(16) $$D(B)=\sum_{i=1}^{s}D_{i}(B)$$

where:

(17) $$D_{i}(B)=\left\{ \begin{array}{l} w_{i\;}if\;T_{i}\in {ℑ}_{i}\\ 0\;else \end{array}\right.$$

The patch *B* is considered as flood (18) if the weighted vote is greater than 0.8 from the sum of all weights (the maximum of *D*):

(18) $$B\in F\;if\;D(B)\geq 0.8\cdot (\sum_{i=1}^{s}w_{i})$$

where 0.8 is an experimentally chosen threshold.

Inside of the analyzed image, a segmentation process is done with the aid of the detected flood patches. For visualization purposes, the flood boxes are marked with white. With the patches from an image, an associate matrix *BMP* is obtained. Each patch corresponds to an element in *BMP*; so, for an image dimension of 4000 × 6000 pixels and a patch of 50 × 50 pixels, then the *BMP* matrix dimension is dim *BMP* = 80 × 120. If the number of marked boxes is *n*, then the percentage of flood zone in the analyzed image is *PF* (22):

(19) $$PF=\frac{n}{\dim BMP}\times 100\;[\%]$$

#### 2.3.3. Algorithm for Flood Detection 

The proposed algorithm has two phases: the Learning Phase—Algorithm 1 and the Mission Phase—Algorithm 2.
**Algorithm 1:** Learning Phase **Inputs:** Learning patches (40 patches for feature selection—set 1 and 100 patches for weight establishing—set 2), set of feature to be investigated;**Outputs:** Selected features *T_i_*, the weights for selected features *w_i_*, and the intervals ${ℑ}_{i}$, *i* = 1, …, 6.  For each patch of the first set: 1. Image decomposition on color channels (*R*, *G*, *B*, *H*, *S*, *V*) of patches; 2. Reject noise with median local filter; 3. Calculate the features: *Im*, *Con*, *En*, *Hom*, *Ent*, *Var*, *Dm* and *L* on color channels; 4. Until end of set 1; 5. Calculate the Confidence Indicator *CI_i_* for each feature based on Equation (11); 6. Feature selection: *T_i_*, *i* = 1, …, 6; 7. Determine the intervals for flood class representative ${ℑ}_{i}$, Equations (13) and (14)  For each *Ti*: 8. Calculate the confusion matrices *CM_i_* from the set 2; 9. Calculate the weights *w_i_*, *i* = 1, …, 6; Equations (15) and (17) 10. Return {*T_i_*, *w_i_*}.
**Algorithm 2:** Classification Phase **Inputs:** Images to be analyzed, Selected features *T_i_*, the weights for selected features *w_i_*, and the intervals ${ℑ}_{i}$, *i* = 1, …, 6;**Outputs:** Segmented images and percent of flooded areas  For each image *I*: 1. Image decomposition in small non-overlapping patches (50 × 50 pixels);  For each patch *B* 2. Calculate the selected features *Im_R_*, *Con_HH_*, *En_HS_*, *Hom*_HH_, *Dm*_G_ and *L_R_*; 3. Calculate *D_i_*(*B*); 4. Patch classification based on voting scheme (18); 5. Until end of patches from image *I_i_*; 6. Create the matrix of patches for each feature; 7. Noise rejection based on local median filter in matrices of patches; 8. Create the final matrix of patches based on voting scheme; 9. Create segmented image; 10. Calculate the percent of flooded area from image with Equation (19); 11. Until end of images to be analyzed; 12. Return the segmented images and percent of flooded area.

**Figure 6:**
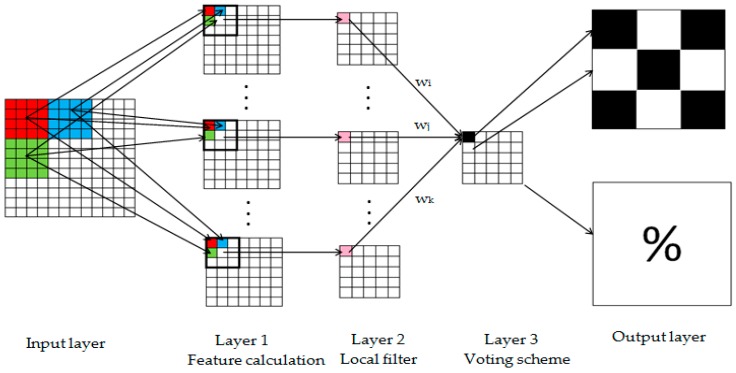
The neural network for the mission phase.

Algorithm 1 is executed only once, at the beginning of the mission, while Algorithm 2 runs continuously throughout the mission. Both are implemented in deep neural networks (DNN). The DNN for Algorithm 2 is presented in Figure 6 and contains, besides the input and output layers, other three layers. 

Layer 1 is dedicated to simultaneously calculate the features of patches and create the corresponding binary matrices of patches. Layer 2 is dedicated to local filtering of matrices from Layer 1, in order to eliminate the noise from BMP. Layer 3 creates the final BMP by voting scheme. Finally, the Output layer provides the segmented image and the relative flood size. 

## 3. Experimental Results 

For experimental results we used a UAV, designed, as coordinator, by University POLITEHNICA of Bucharest, MUROS project [35]. The main characteristics and technical specifications of UAV MUROS, as mobile node for image acquisition, are presented in Table 3. 

**Figure 7:**
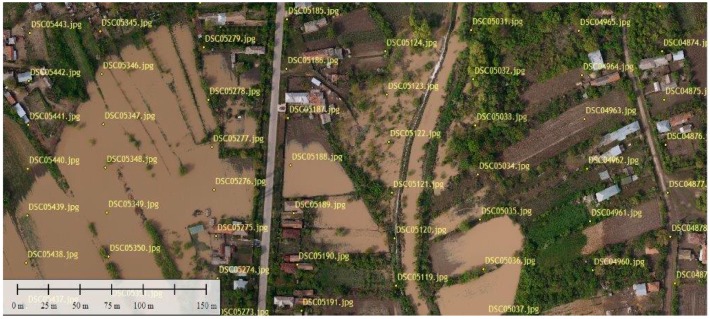
Image created from acquired images (with yellow ID) without overlapping or gaps. The image was generated with Agisoft Photoscan Professional Edition (www.agisoft.com).

To evaluate the algorithms presented in Section 2, an image dataset of a flooded area was gathered with MUROS. The photographs have been captured along a path generated as in Section 2, with distances between lines *d* = 75 m and height of flight de = 100 m (wind strength was considered to be negligible). The portion from the orthophotoplan of an application near Bucharest, during a flood, is presented in Figure 7. The images analyzed with the algorithm described in Section 2 are marked with the specified IDs. 

**Figure 8:**
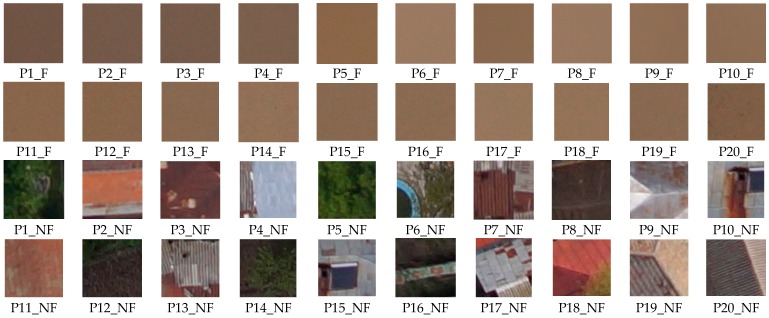
Patches for establish the flood signature (Pi_F as patch with flood and Pj_NF as non flood patch).

In the learning phase, for patch signature determination *T*, the first set of 40 patches of dimension 50 × 50 pixels (20 patches for flood and 20 for non-flood), manually selected, was used (Figure 8). From this set, a cluster of 20 patches containing only flood (*PF*) are considered to form the prototypes for the class “flood” (*F*) and 20 patches, containing non flood elements (*PNF*), e.g., buildings and vegetation, are considered for the class “non-flood” (*NF*).

The results obtained in the learning phase (Table 4) show that the selected features (with *CI* criterion) are: *ImR*, *ConHH*, *HomHH*, *EnHS*, *DmG* and *LR*, where R, G, H, and S are the components of the color spaces. Thus, features on different types (first order statistics, second order statistics and fractal), on different channel color are selected. If *CI* falls below 0.80, then the accuracy can also decrease. It must be mentioned that the list of selected features can be changed in the learning phase, upon the requirements of the application. The fractal dimension was calculated by means of FracLac [42] plug-in of ImageJ and the features extracted from co-occurrence matrix were computed using MATLAB software. In Table 4, the values marked with * are those that are not within the corresponding confidence intervals.

Next step is the calculation of the confusion matrices for the selected features (Table 5). To this end, we used the second set (100 patches) for the learning phase, which contains 50 patches marked as flood (actually) and 50 patches marked as non-flood. From the confusion matrices we calculate the weights *w_i_* which will be used further for patch classification.

So, the signature of the patch is:

T=[T1,T2,T3,T4,T5,T6]=[ImR,ConHH,HomHH,EnHS,DmG,LR]

and the associate weights are:

$$\left[w_{1},w_{2},w_{3},w_{4},w_{5},w_{6}]=[0.95,\;1,\;1,\;1,\;0.90,\;0.95\right]$$

The representative of the class *F* is:

$$\begin{array}{l} \left[{ℑ}_{1},{ℑ}_{2},{ℑ}_{3},{ℑ}_{4},{ℑ}_{5},{ℑ}_{6}\right]=\\ \left[\left[0.418;\;0.535],[0.994;\;1.002],[-0.004;\;0.011],[0.896;\;0.951],[2.605;\;2.709],[0.344;\;0.502\right]\right] \end{array}$$

**Figure 9:**
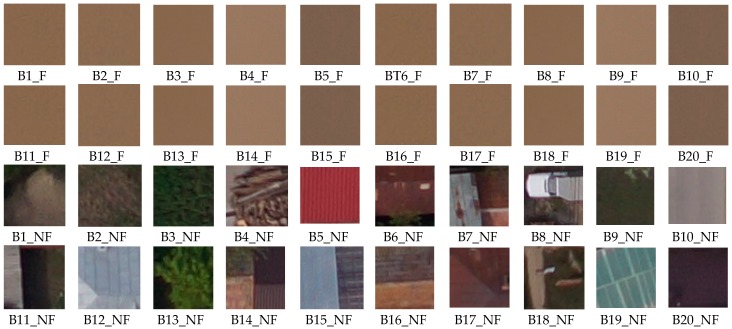
Patches for establish the weight signature (Bi_F as patch with flood and Bj_NF as non flood patch).

**Figure 10:**
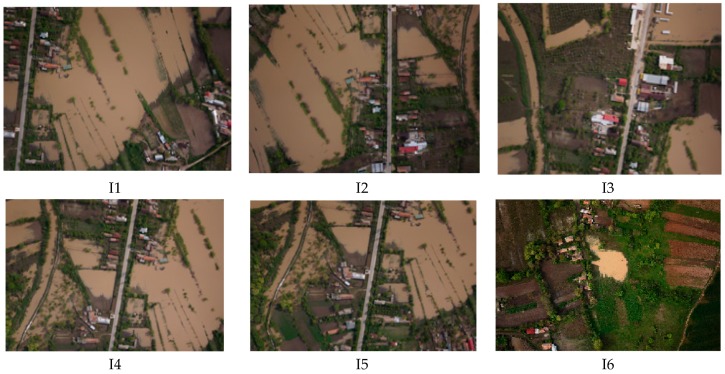
Images acquired by UAV MUROS to be evaluate for flood detection.

**Figure 11:**
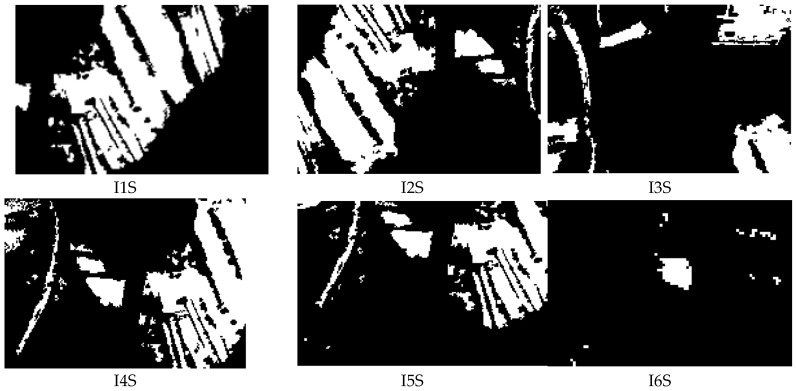
Images segmented for flood evaluation. White—flooded areas; black—non flooded areas.

**Figure 12:**
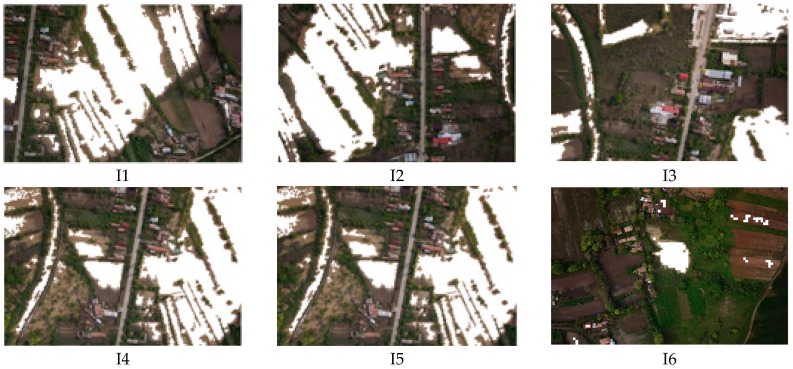
The overlap of RGB images with the segmented images.

In order to analyze the performances of the algorithm for flood detection, a set of 50 images with flood was investigated (see orthophotoplan from Figure 7). Random patches of flood and non flood types (Figure 9) are classified based on the voting scheme and the results are presented in Table 6. Here, *D*(*B*) is calculated as in (16) and compared with maximum value of $0.8\cdot (\sum_{i=1}^{s}w_{i})=5.59$ as in (17). For example, patches B6_F and B10_F with flood are wrongly classified as non flood. For the mission phase, an example of 6 images used for the algorithm test is presented in Figure 10 and the result of the segmentation, in Figure 11. Figure 12 overlaps the RGB images with masks generated by segmented images.

The random errors of the classification process are characterized by sensitivity, specificity, and accuracy [10,43] which are calculated in Table 7, where: *TP* is the number of true positive cases, *TN* is the number of true negative cases, *FP* is the number of false positive cases, and *FN* is the number of false negative cases. In [12] an accuracy of 87% is obtained using RGB information and six texture features (fixed) extracted from gray level co-occurrence matrix. Our method uses selected features (selected by a performance criterion at the beginning of the segmentation operation) on color channels (chromatic co-occurrence matrix and fractal type) and the accuracy was of 98.1%.

## 4. Discussion

Because we considered only complete flooded boxes, the approximation will be underestimated. Similarly, if mixed boxes are considered, an over approximation will be obtained. Table 8 presents the number of patches considered as *F* and the corresponding percent of flooded area for each images. The main cause was the patches from the contour of flooded which appear as mixed ones. Further studies will consider the decomposition of these patches in boxes increasingly small.

On the other hand, by properly choosing textural features on color channels and patch dimension, the proposed algorithm can be extended to more classes like: road, vegetation, buildings, etc. Combining thermal camera with video, the system is able to detect possible persons in difficulty and to monitor the rescue operation. In this case, a flexible and dynamic strategy for trajectory design is necessary. Also the collaboration between the mobile nodes (UAVs) will improve the mission efficiency. The algorithms used for trajectory design minimize total path length while in the same time passing through (or within predefined distance) of a priori given way-points. In further work we plan to: (i) reconfigure trajectories on the fly such that the flooded areas are covered efficiently; and (ii) partition the workload of the UAVs such that total time/effort is minimized (for now we simply divide the area of observation into disjoint regions, one per each UAV).

## 5. Conclusions 

The paper presented a comprehensive system and methodology for the detection and segmentation of flooded areas in a pre-determined zone. The contributions are focused on two important objectives: the planning of an optimal trajectory to cover the area under investigation and the image processing required to detect and to evaluate the flood spread. For the first the novelty lies in the analysis and computation of an optimal path covering the area of interest and for the second, the novelty lies in combining the information for different color channels with information about spatial pixel distribution obtained from chromatic co-occurrence matrix and mass fractal dimension. First, the paper studied a typical photogrammetry problem through the prism of control and optimization theory. That is, for a given polyhedral region which has to be covered by parallel lines (along which photographs are taken) we have given both an estimation of the required number of photographs and provided a minimum-length path covering the area. For the latter case we formulated a constrained optimization problem where various constraints and parameters were considered in order to obtain a minimum-length path. We took into account the maximum distance between consecutive lines and turn conditions (such that the UAV is guaranteed to follow the interior lines). We have also discussed the path generation problem in the presence of wind and for regions with non-convex shapes. Second, a methodology for the detection, segmentation and evaluation of flooded areas from the acquired images was presented. A color co-occurrence matrix was introduced and some efficient features. Furthermore, we illustrated that fractal type features on color component improve the local classification process on flooded and non-flooded boxes. The algorithm was tested on a large number of sub-images and the results showed good performances. We conclude that, by including the color information to texture analysis, by selection of feature based on maximum criterion and by using the fractal techniques, the accuracy of the detection of flooded boxes was increased up to 99.12%.

## Figures and Tables

**Table 1:** MUROS UAV—Abbreviations and functionality.

Abbreviation/Module Name	Function
FMCU Flight and Mission Control Unit	-Coordinates the flight mission; -Provides the platform’s stability and quick response in case of disturbances that may deflect the drone from its pre-defined route or its removal from the flight envelope; -Allows for manual piloting by an operator on the ground; -Implements the automated low-level control loops which assure path tracking.
AHRS Attitude and Heading Reference System	-Provides information for an autonomous flight; -Contains the sensor subsystem composed of static and dynamic pressure sensors for speed measurement (ADXL352), accelerometer (ASDXRRX005PD2A5), magnetometer (HMC5983), altimeter (MPL3115A2) and gyroscope (ADXRS450); -Data provided by AHRS are used by FMCU.
SU Safety Unit	-Assures the permanent monitoring of the signals sent by other units and interprets the error signals received; -Taking into account the fault-tree and the reported error, the SU may decide the future functioning of the UAV. Thus, it can decide to continue the mission, to return to the launching point or the designated retrieval point, or, as a last step, to deploy the parachute.
PU Power Unit	-Assures the electrical power to the other components of the UAV, especially to the propulsion motor; -Contains power sources and a storage balance sensor used to equilibrate the energy consumed.
VD Video Datalink	-Sends video data from the camera (PS) to the ground (via the GDT, to the GCS). It contains a modem RF (TXR) and a power amplifier RFA.
TD Telemetric Datalink	-Assures a duplex communication for both transmission and reception of telemetry data. It has a structure similar to the VD.
Payload Working load (payload)	-Has a dedicated CPU for device retracted; -Provides high resolution imagery or video HD; -Based on a gyro-stabilized mechanism.
GDT Ground Data Terminal	-Antenna based tracking system; -The operational range is extended by using multiple ground data terminals; -Radio and Internet connections.
GCS C Ground Control Station Coordinator	-Is the main component of the system; -Has a friendly user interface for operational purposes; -Internet connection with GDTs and IPU.
GCS L Local Ground Control Station	-Optional -Transfer the control to operational field for each UAV.
CSU Control for Servomotor Unit	-Ensures the control of the electric actuators; -Provides a feedback on their state.
CRU Control Radio Unit	-Ensures the radio data transmission to and from GDT: telemetry, video/images and control.
DESP Data Exchange & Signal Processing	-Data exchange between GCS and UAV via GDT; -Encoding/ decoding of video data; -Interface with Ethernet IP (ETH).
SPTU Servo Pan Tilt Unit	-Transmission of control to the payload servomotors.
PFCT PC for Flight Control and Telemetry	-Is the main module of GCS and is based on a CPU.
ETH Switch Ethernet	-Ensures the data transmission at distance.
RC Radio Control	-Ensures the control transmission to the GDT.
LL Launcher Link	-Ensures the interface of GCS with the launcher; -Transmits the launch command.
SL Safety Launcher Module	-Assures the start of UAV propulsion, if the speed launch is correct.
IPU Imge Processing Unit	-Processes the images for flood detection -Estimate the size of flooded areas.
ORT Ortho-rectified module	-Creates the ortho-rectified images.
PLAN Ortho-photoplan module	-Creates the ortho-photoplan.
LP Learning module	-Establishes the patches for feature selection; -Establishes the class representatives and features for patch signatures.
CP Clssification module	-Divides the image in patches; -Classifies the patches as flood and non flood.
DE Flood detection and estimation module	-Creates the segmented images -Estimates the flooded area (in percent).
WiFi Module for WiFi communication	-Assures WiFi communication.

**Table 2:** Analyzed features.

Energy	$En_{d}=\sum_{i=1}^{K}\sum_{j=1}^{K}C_{d}{\left(i,j\right)}^{2}$	Contrast	$Con_{d}=\sum_{i=1}^{K}\sum_{j=1}^{K}{\left(i-j\right)}^{2}C_{d}(i,j)$
Entropy	$Ent_{d}=-\sum_{i=1}^{K}\sum_{j-1}^{K}C_{d}(i,j)\cdot \log_{2}[C_{d}(i,j)]$	Correlation	$\sum_{i=1}^{K}\sum_{j=1}^{K}\frac{i\cdot j\cdot C_{d}(i,j)-\mu_{x}\mu_{y}}{\sigma_{x}\sigma_{y}}$
Homogeneity	$Hom_{d}=\sum_{i=1}^{K}\sum_{j=1}^{K}\frac{C_{d}\left(i,j\right)}{1+\left|i-j\right|}$	Mean intensity	Im=1M×R∑i=1R∑j=1MI(i,j)
Variance	$\sum_{i=1}^{K}\sum_{j=1}^{K}{\left(i-\mu \right)}^{2}\cdot C_{d}(i,j)$	LBP Histogram	$H=\left[H_{0},H_{1},\ldots ,H_{n-1}\right]$
Mass fractal dimension	$Dm=\frac{\log(\sum_{u}\sum_{v}n_{r}(u,v))}{\log r}$	Lacunarity	$L(r)=\frac{\sum_{n}n^{2}\cdot \;P(n,r)}{[\sum_{n}n\cdot P(n,r){]}^{2}},\;n=\sum_{u}\sum_{v}n_{r}(u,v)$

**Table 3:** MUROS UAV—Characteristics and technical specifications.

Characteristics	Technical Specifications
Propulsion	Electric
Weight	15 kg
Wingspan	4 m
Endurance	120 min
Operating range	15 km in classical regime and 30 km in autopilot regime
Navigation support	GIS
Navigation	manual/automatic
Communication	antenna tracking system
Payload	retractable and gyro-stabilized
Mission	Planning software
Recovery system	Parachute
Maximum speed	120 km/h
Cruise speed	70 km/h
Maximum altitude	3000 m
Maximum camera weight	1 kg
Camera type	Sony Nex7, objective 50 mm, 24.3 megapixels, 10 fps
Parameters for flood detection	Flight speed of 70 km/h and flight level 300 m
Typical applications	Monitoring of critical infrastructures, reconnaissance missions over the areas affected by calamities (floods, earthquakes, fires, accidents, etc.), camera tracking, photography and cartography

**Table 4:** The selected features, their confidence indicators and the representatives for the class *F*.

Patch	*ImR*	*HomHH*	*ConHH*	*EnHS*	*DmG*	*LR*
P1_F	0.460	0.999	0.001	0.916	2.667	0.445
P2_F	0.472	0.997	0.003	0.921	2.690	0.432
P3_F	0.484	0.998	0.001	0.911	2.665	0.387
P4_F	0.504	0.998	0.007	0.932	2.641	0.455
P5_F	0.478	0.999	0.007	0.926	2.668	0.485
P6_F	0.488	0.996	0.001	0.919	2.643	0.415
P7_F	0.475	0.996	0.008	0.915	2.639	0.395
P8_F	0.485	0.997	0.002	0.912	2.635	0.401
P9_F	0.506	0.999	0.001	0.928	2.664	0.413
P10_F	0.443	0.998	0.001	0.926	2.671	0.398
P11_F	0.433	0.995	0.001	0.934	2.648	0.446
P12_F	0.486	0.997	0.002	0.924	2.685	0.432
P13_F	0.479	0.999	0.003	0.909	2.645	0.457
P14_F	0.502	0.996	0.003	0.914	2.654	0.395
P15_F	0.477	0.996	0.001	0.921	2.675	0.438
P16_F	0.491	0.997	0.002	0.929	2.632	0.442
P17_F	0.465	0.999	0.007	0.941	2.643	0.413
P18_F	0.451	0.998	0.005	0.937	2.642	0.428
P19_F	0.462	1.000	0.006	0.938	2.685	0.391
P20_F	0.498	0.999	0.004	0.917	2.650	0.394
$m_{i}$	0.476	0.997	0.003	0.923	2.635	0.423
${ℑ}_{i}$	**[0.418; 0.535]**	**[0.994; 1.002]**	**[−0.004; 0.011]**	**[0.896; 0.951]**	**[2.605; 2.709]**	**[0.344; 0.502]**
P1_NF	0.161	0.195	0.392	0.415	2.601	0.177
P2_NF	0.302	0.176	0.591	0.580	2.581	0.182
P3_NF	0.226	0.187	0.560	0.602	2.592	0.164
P4_NF	0.201	0.588	0.621	0.604	2.557	0.161
P5_NF	0.241	0.576	0.399	0.424	2.569	0.345 *
P6_NF	0.151	0.192	0.581	0.522	2.590	0.194
P7_NF	0.160	0.184	0.395	0.589	2.583	0.176
P8_NF	0.215	0.177	0.581	0.449	2.596	0.167
P9_NF	0.210	0.583	0.632	0.608	2.562	0.155
P10_NF	0.151	0.593	0.481	0.625	2.568	0.174
P11_NF	0.356	0.192	0.492	0.519	2.656 *	0.255
P12_NF	0.152	0.201	0.353	0.450	2.592	0.162
P13_NF	0.169	0.171	0.372	0.561	2.590	0.175
P14_NF	0.211	0.581	0.367	0.382	2.577	0.145
P15_NF	0.205	0.544	0.624	0.613	2.573	0.198
P16_NF	0.174	0.193	0.368	0.402	2.590	0.207
P17_NF	0.195	0.576	0.634	0.634	2.562	0.184
P18_NF	0.382	0.476	0.587	0.596	2.606 *	0.195
P19_NF	0.421 *	0.425	0.456	0.545	2.584	0.198
P20_NF	0.203	0.543	0.429	0.512	2.597	0.178
$\eta (\lambda_{i})$	1	0	0	0	2	1
η(PNF)	20	20	20	20	20	20
*CI*	0.95	1	1	1	0.90	0.95

*: The values are not within the corresponding confidence intervals.

**Table 5:** The confusion matrices and the resulting weights for the selected features.

*ImR* = *T*_1_	*HomHH* = *T*_2_	*ConHH* = *T*_3_	*EnHS* = *T*_4_	*DmG* = *T*_5_	*LR* = *T*_6_
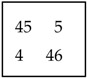	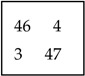	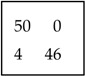	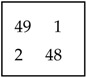	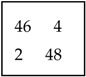	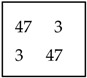
*w*_1_ = 0.91	*w*_2_ = 0.93	*w*_3_ = 0.96	*w*_4_ = 0.97	*w*_5_ = 0.88	*w*_6_ = 0.94

**Table 6:** Some experimental results concerning the patch classification based on voting scheme. Gray rows mean wrong classification.

Patch (Actual)	*ImR*/*D*(*B*_1_)	*HomHH*/*D*(*B*_2_)	*ConHH*/*D*(*B*_3_)	*EnHS*/*D*(*B*_4_)	*DmG*/*D*(*B*_5_)	*LR*/*D*(*B*_6_)	*D*(*B*)/*F*,*NF* *T* = 0.8 × 5.59
B1_F	0.494/0.91	0.996/0.93	0.001/0.96	0.942/0.97	2.661/0.88	0.372/0.94	5.59/F
B2_F	0.506/0.91	0.998/0.93	0.003/0.96	0.9340.97	2.637/0.88	0.421/0.94	5.59/F
B3_F	0.457/0.91	0.999/0.93	0.006/0.96	0.9610.97	2.643/0.88	0.446/0.94	5.59/F
B4_F	0.464/0.91	0.999/0.93	0.005/0.96	0.9160.97	2.701/0.88	0.497/0.94	5.59/F
B5_F	0.515/0.91	0.997/0.93	0.004/0.96	0.9520.97	2.621/0.88	0.480/0.94	5.59/F
B6_F	0.398/0	0.995/0.93	0.021/0	0.899/0.97	2.587/0	0.346/0.94	2.84/NF
B7_F	0.437/0.91	0.998/0.93	0.003/0.96	0.9190.97	2.678/0.88	0.405/0.94	5.59/F
B8_F	0.493/0.91	0.997/0.93	0.004/0.96	0.9310.97	2.671/0.88	0.417/0.94	5.59/F
B9_F	0.476/0.91	0.995/0.93	0.003/0.96	0.9150.97	2.682/0.88	0.482/0.94	5.59/F
B10_F	0.350/0	0.992/0	0.013/0	0.850/0	2.623/0.88	0.321/0	0.88/NF
B1_NF	0.172/0	0.204/0	0.387/0	0.423/0	2.599/0	0.167/0	0/NF
B2_NF	0.137/0	0.189/0	0.582/0	0.502/0	2.579/0	0.202/0	0/NF
B3_NF	0.224/0	0.526/0	0.353/0	0.412/0	2.564/0	0.327/0	0/NF
B4_NF	0.198/0	0.537/0	0.624/0	0.623/0	2.538/0	0.211/0	0/NF
B5_NF	0.249/0	0.592/0	0.617/0	0.589/0	2.521/0	0.149/0	0/NF
B6_NF	0.335/0	0.213/0	0.457/0	0.501/0	2.599/0	0.268/0	0/NF
B7_NF	0.186/0	0.555/0	0.602/0	0.654/0	2.556/0	0.172/0	0/NF
B8_NF	0.139/0	0.185/0	0.366/0	0.573/0	2.572/0	0.161/0	0/NF
B9_NF	0.231/0	0.593/0	0.401/0	0.438/0	2.569/0	0.339/0	0/NF
B10_NF	0.391/0	0.821/0	0.009/0.96	0.722/0	2.651/0.88	0.311/0	1.84/NF

**Table 7:** Statistic for flooded area in images: 1000 pathces (500—flood, 500—non flood).

*TP*	*TN*	*FP*	*FN*	Sensitivity	Specificity	Accuracy
486	495	5	14	97.2%	99%	98.1%

**Table 8:** Percent of flooded area.

Images	IS1	IS2	IS3	IS4	IS5	IS6
Percent	32.88	32.79	16.85	28.07	21.57	2.44
No. patches	3156	3148	1617	2695	2071	234

## Appendix A. The Pseudo Code (Matlab) for the Proposed Algorithm for Computing CCM between Spectral Components A and B

BEGIN Compute(A, B, xdimension, ydimension, n) 
        A = normalize(A,n); 
        B = normalize(B,n); 
        Initialize result as a n × n array of 0; 
        bExtended = extendMatrix(B, xdimension, ydimension); 
        offset = calculateOffset([xdimension, ydimension]); 
        for i = 0 to n 
              for j = 0 to n 
                    positions = searchAppearences(A, i); 
                    if positions ! = null 
                         for k = 0 to positions[0].length
                         bRow =positions[0][k] +offset[1] + xdimension; 
                         bCol = positions[1][k] + offset[2] + ydimension; 
                         if bExtended[bRow][bCol] == j 
                              result[i][j] ++; 
        return result; 
        END 
        BEGIN calculateOffset(offsetIn) 
        for i = 1 to offsetIn.length 
              if offsetIn(i) >= 0 
                    offsetOut(i) = 0; 
              else offsetOut(i) = abs(offsetIn(i)); 
        END 
        BEGIN searchAppearences(A, x) 
        count = countAppearences(A, x); 
        initialize positions as a 2 x count array; 
        if count == 0 
             return null; 
        int k = 0; 
        for i = 0 to rows 
        for j = 0 to cols 
              if A[i][j] == x 
                    positions[0][k] = i; 
              positions[1][k] = j; 
              k++; 
        return positions; 
        END 
        BEGIN countAppearences(A, int x) 
        count = 0; 
        for i = 0 to rows 
              for j = 0 to cols 
                    if A[i][j] == x 
                          count++; 
        return count; 
        END 
        BEGIN extendMatrix(A, noRows, noCols) 
        initialize result as an array (A.length + abs(noRows))x(A[0].length + abs(noCols)); 
        offset = calculateOffset([rowsNo, colsNo]); 
        for i = 0+offset[1] to A.length+offset[1] 
              for j = 0+offset[2] to A[0].length+offset[2] 
                    result[i][j] = A[i][j]; 
        for i = A.length to A.length + noRows 
              for k = 0 to A[0].length + noCols 
                    result[i][k] = −1;
        for i = A[0].length to A[0].length + noCols 
              for k = 0 to A.length + noRows 
                    result[k][i] = −1; 
        return result; 
        END
		
